# Zebrafish Embryos to Profile Nano(bio)materials: A Modular Platform for Developmental Toxicity, Neurotoxicity, and Inflammation‐Regeneration Assays

**DOI:** 10.1002/cpz1.70403

**Published:** 2026-06-13

**Authors:** Cinzia Bragato, Alessandra Lama, Greta Carbone Faccin, Johan George Varghese, Rossella Bengalli, Patrizia Bonfanti, Anita Colombo, Maurizio Gualtieri, Paride Mantecca

**Affiliations:** ^1^ POLARIS Research Laboratory, Department of Earth and Environmental Sciences University of Milano‐Bicocca, Milan Metropolitan City of Milan Italy

**Keywords:** inflammation model, lipopolysaccharide (LPS), macrophages, nanotoxicology, nanomaterials, neutrophils

## Abstract

Zebrafish embryos provide a rapid, low‐cost in vivo platform to profile the developmental, neurotoxic, and immunomodulatory effects of engineered nano(bio)materials within a single vertebrate system. This article presents a modular workflow that integrates standardized fish embryo acute toxicity (FET) testing, chemically assisted dichlorination, inflammatory challenge, and regeneration assays to evaluate both bio‐based and synthetic nanoparticles. First, embryos are exposed to nanomaterials under FET conditions and subjected to quantitative morphometric analysis to establish sublethal concentration ranges and detect subtle growth defects. Sensitivity to nano(bio)material exposure is enhanced by a pronase‐based dechorionation procedure, which removes the chorion to promote direct particle–tissue contact while maintaining acceptable baseline viability. Immunoinflammatory responses are probed using two complementary modules: microinjection of *Pseudomonas aeruginosa* lipopolysaccharide (LPS) into the yolk sac to induce acute systemic inflammation and caudal fin wounding to elicit a localized inflammatory and regenerative response. Across these models, we combine qPCR panels of cytokine and *Wnt/β‐catenin* pathway genes with Sudan Black B staining of neutrophils and neutral red vital staining of macrophages, providing accessible cellular and molecular readouts in wild‐type embryos. Together, these protocols constitute a harmonized, technically accessible pipeline that enables comparative safety and bioactivity profiling of diverse nano(bio)materials and supports their assessment within safe‐and‐sustainable‐by‐design frameworks. © 2026 The Author(s). *Current Protocols* published by Wiley Periodicals LLC.

**Basic Protocol 1**: Nanoparticle preparation and suspension, and embryo dechorionation procedure (pronase solution)

**Basic Protocol 2**: Sudan Black B staining protocol

**Basic Protocol 3**: Neutral red staining protocol

**Basic Protocol 4**: LPS microinjection protocol

**Basic Protocol 5**: Wound inflammation model generation protocol

## INTRODUCTION

Zebrafish embryos provide a rapid and low‑cost in vivo platform for assessing engineered nanomaterials (NMs), capturing developmental, neurobiological, and innate immune endpoints within a single organism. Here, we propose a methodological framework for the biocompatibility and adverse effects of silica‐based NMs, based on experimental approaches and previously published results (Bragato et al., 2024a). Studies were performed using bio‑based and synthetic silica nanoparticles (SiO_2_‑NPs), lignin nanoparticles (LigNPs) and phenolated lignin nanoparticles (PheLigNPs) (Bragato et al., 2024a, 2024b), and *Penicillium expansum* filtrate, combining standardized embryo exposure, neurodevelopmental imaging, two complementary inflammatory–regeneration assays, and macrophage observation. The pipeline is designed to be technically accessible, reproducible, and readily adaptable to diverse NM classes.

The **first module** implements the FET test [OECD (2025), Test No. 236] to assess the basic developmental compatibility of SiO_2_‑NPs. Briefly, embryos are maintained in fish embryo solution (ES) and exposed from the early cleavage stage to a concentration range of SiO_2_‑NPs (e.g., 0.1–100 µg/ml) under static conditions, monitoring mortality and sublethal defects up to 96 hpf. At 96 hpf, morphometric parameters, including body length, eye area and diameter, interocular distance, head width, and yolk sac area, are quantified to detect subtle growth alterations. In our experience, bio‑based SiO_2_‐NPs, derived from rice husk and commercial fumed SiO_2_‐NPs, showed negligible acute toxicity in embryos with chorion across the tested range, but morphometrics and immune readouts allowed discrimination of more subtle effects (Bragato et al., 2024a).

To enhance sensitivity to NM exposure, the same FET framework can be applied to chemically dechorionated embryos [ISO/TS 22082:2020(E)].

This is based on the use of pronase, an enzyme mix widely used in molecular biology and zebrafish research for protein digestion and embryo manipulation, derived from the soil bacterium *Streptomyces griseus* (Trop and Birk, 1970).

Despite being a common method used for dechorionation of zebrafish embryos, protocols show considerable variability in both the concentration of pronase used and the length of exposure, with little harmonization across studies (Hasegawa et al., 2023; Henn & Braunbeck, 2011; Mandrell et al., 2012). We propose an easy‐to‐handle manual protocol that results in an acceptable mortality rate at early developmental stages.

Embryos at early gastrula or epiboly stages are incubated briefly (∼30 s) in pronase (1 mg/ml; working solution B, see Basic Protocol 1) at room temperature, gently agitated, and then extensively washed (at least 6–7 changes of embryo medium) to remove enzyme and detached chorions. Any remaining chorions can be mechanically opened under a stereomicroscope. This approach typically results in a background mortality of ∼20%–25%, which should be accounted for in both experimental design and controls; importantly, this mortality is consistently equivalent between vehicle‐treated control embryos and nanoparticle‐exposed groups and does not confound group comparisons. In our published studies (Bragato et al., 2024a, 2024b), surviving pronase‐dechorionated control embryos showed no significant increase in spontaneous morphological defects relative to intact chorion controls across a panel of morphometric endpoints (body length, eye area, interocular distance, yolk sac area, and cardiac morphology), confirming that the surviving population is developmentally normal and suitable for downstream assays, but enables direct contact between the NM suspension and the embryonic surface. For SiO_2_‐NPs that tend to agglomerate on the chorion, dechorionation can be essential to evaluate true particle–tissue interactions rather than chorion trapping (Bragato et al., 2024a, 2024b).

In addition to lethal and malformation effects, especially for insoluble NMs, considering their mild acute toxicity and potential high persistence, such as for the newly developed bio‐based ones, additional investigations are worthy of possible subacute effects. In this perspective, neurodevelopmental and immunomodulation responses should be prioritized.

Neurodevelopmental effects are assessed in a **second module** using Tg(‐3.1*neurog1*:EGFP) embryos as a live readout of primary neurogenesis (Blader et al., 2003; McGraw et al., 2008). Transgenic embryos are mechanically dechorionated at the end of somitogenesis (18–20 hpf) and exposed to selected concentrations of SiO_2_‑NPs (e.g.,100–200 µg/ml) for 96 h, maintaining standard FET conditions. At 96 hpf, embryos are anesthetized and imaged on an epifluorescence stereomicroscope, acquiring whole‑mount GFP images under fixed exposure and magnification settings. The GFP‑positive area and/or mean intensity in the *neurog1* expression domains are quantified using ImageJ/Fiji (https://imagej.net/Fiji) to detect alterations in spatial patterning or signal strength. In our SiO_2_‑NP experiments, neither the bio‑based nor the commercial particles produced measurable changes in *neurog1*‑driven GFP expression, supporting the use of this module as a sensitive yet straightforward screen for neurodevelopmental disruption [Bragato et al., 2024a].

Considering the relevance of inflammation in immune response against toxic or irritating stimuli and the importance of this process in damaging tissues when deregulated [Bender et al., 2025], the **third module** introduces an acute inflammation assay.

A variety of sophisticated approaches have been established to study bio‐NM‑induced inflammation in zebrafish embryos, including the use of transgenic fluorescent reporter lines, high‑resolution microscopy, and molecular analyses such as qPCR or transcriptomics (d'd'Alençon et al., 2010; Gillies et al., 2022; Johnston et al., 2025; Muttalik et al., 2024; Pensado‐Lòpez et al., 2021; Rizzo et al., 2013). However, these methods require specific animal lines, advanced imaging platforms, or dedicated biochemical facilities, which may limit their widespread adoption, especially in laboratories primarily focused on ecotoxicology or material screening. Therefore, there is a clear need for a user‑friendly and standardized protocol that can be implemented in wild‑type embryos using only basic equipment, enabling reproducible assessment of the inflammatory effects of NMs even in settings without transgenic zebrafish or specific infrastructure.

We propose a method providing such a practical tool, lowering the technical threshold while maintaining biologically relevant inflammatory readouts, specifically for testing bio‐based NM effects.

Inflammatory responses are assessed using a combination of vital dyes and molecular endpoints that are compatible with wild‑type zebrafish embryos and standard laboratory equipment. Neutrophil recruitment and distribution are visualized in fixed embryos by Sudan Black B staining, which labels myeloperoxidase‑positive neutrophil granules.

Macrophages are evaluated by neutral red vital staining, exploiting their phagocytic uptake of the dye to reveal macrophage populations in vivo.

As a positive control for inflammation, embryos are injected with lipopolysaccharide (LPS), a well‑established trigger of innate immune activation in zebrafish (Novoa et al., 2009; Yang et al., 2014), which we slightly modified for a more focused purpose.

In parallel, the expression of a panel of pro‑inflammatory genes is quantified by qPCR (Supplementary information), providing a sensitive molecular readout that complements cellular staining and allows detection of more subtle bio‐based NM‑induced immune effects.

Neutrophil recruitment is quantified using Sudan Black B staining. Fixed embryos (typically 72–96 hpf) are stained in Sudan Black working solution, thoroughly destained in 70% ethanol, and rehydrated in PBST before imaging. Neutrophils appear as darkly stained cells concentrated at injury sites (e.g., caudal fin wound) or in hematopoietic regions such as the caudal hematopoietic tissue (CHT). The stained area or particle number within a standardized region of interest is quantified using threshold‑based analysis and particle counting in Fiji, yielding an objective measure of neutrophil response to both insult and NM exposure. In SiO_2_‑NP studies, this approach differentiated bio‑based SiO_2_ with minimal impact on neutrophil numbers from commercial SiO_2_ that significantly increased CHT neutrophils at high concentrations (Bragato et al., 2024a). We also performed this staining to evaluate neutrophils after lignin‐based NP exposure. We found that at the tissue level, LigNPs and PheLigNPs do not exacerbate the acute inflammatory episode triggered by the wound (Bragato et al., 2024b).

We evaluate macrophage behavior following inflammatory stimulation, usually observing a robust response in zebrafish embryos, characterized by direct recruitment, activation, and sustained presence after exposure (Zhang et al., 2017). Macrophages typically arrive after the initial neutrophil influx, move more slowly, and accumulate around injured or NM‐exposed regions where they engulf cell debris, microbes, and particles. Live imaging and functional studies have shown that these cells can form a rim along the wound margin and persist longer than neutrophils, thereby influencing both the progression and resolution of the inflammatory reaction (Mathias et al., 2009). At the molecular level, zebrafish macrophages adopt distinct activation states reminiscent of M1/M2 polarization, with early induction of pro‐inflammatory markers such as *tnfα*, *ccl34a.4*, and *cxcl11aa*, followed by a shift toward anti‐inflammatory, pro‐regenerative profiles as healing proceeds. This dynamic behavior makes macrophages a particularly informative endpoint for evaluating the impact of inflammatory stimuli, including bio‐based NMs, in zebrafish embryo assays (Nguyen‐Chi et al., 2015; Rougeot et al., 2019).

Based on literature demonstrating that zebrafish embryos mount a robust innate immune response to Gram‑negative endotoxins (Novoa et al., 2009; Yang et al., 2014), we propose using LPS derived from *Pseudomonas aeruginosa* as a positive control for inflammation in our model. Previous work has shown that zebrafish larvae respond to *P. aeruginosa* LPS with a clear pro‑inflammatory transcriptional profile and increased mortality in an age‑ and dose‑dependent manner, indicating that this endotoxin is efficiently recognized by the innate immune system of zebrafish. LPS from *E. Coli* is used too, even if it is typically preferred for milder, dose‐dependent inflammatory response and sepsis models, compared to *P. aeruginosa* LPS, characterized by a stronger inflammatory response induction (Liu et al., 2024; Yang et al., 2017).

In line with established LPS‑based inflammatory models in zebrafish, where LPS is delivered either by immersion or by microinjection into the yolk sac to induce acute systemic inflammation, we microinjected *Pseudomonas aeruginosa* LPS (strain ATCC 27316, 1 nl of 250 µg/ml) into the apical portion of the yolk of 30 hpf embryos to generate a reproducible acute inflammatory insult. This injection site was selected to minimize mechanical damage and to exploit physiological yolk resorption and proximity to the circulatory system, thereby favoring the systemic distribution of LPS. Under these conditions, LPS‑injected embryos (positive control group) displayed increased expression of key inflammatory mediators, including *tnfα*, *nfkb2*, *il8*, *il6, il1*, *nfkbiaa*, and *ccl34a.4*, confirming the successful induction of an acute inflammatory state that provides a reliable reference benchmark for evaluating the modulatory effects of lignin‑based nanoparticles.

At 96 hpf, pools of embryos are processed for RNA extraction and qRT‑PCR, using a panel of inflammation‑related genes including *tnfα*, *nfkb2*, *il8*, *il6*, *il1β*, *nfkbiaa*, and *ccl34a.4*, normalized to housekeeping genes such as *lsm12b* or *mobk13*. This setup allows one to distinguish direct NM effects from their capacity to modulate a defined inflammatory insult, for example, by dampening or amplifying LPS‑induced cytokine expression (Bragato et al., 2024b).

The **fourth module** models injury‑induced inflammation and tissue regeneration through caudal fin wounding (Keightley et al., 2014; Miskolci et al., 2019). Anesthetized embryos at 48 hpf are positioned under a stereomicroscope, and a clean, reproducible cut is made in the caudal fin with a fine needle. Wounded embryos are then divided into groups exposed or not exposed to the test NMs (e.g., 100 µg/ml LigNPs or PheLigNPs) and maintained at 27–28°C in ES. Regenerative growth of the fin is monitored over 48–72 h by repeated bright‑field imaging, and the regrowth area is quantified on a representative picture by ImageJ. In parallel, gene expression of the same cytokine panel as above, along with *Wnt/β‑catenin* pathway markers (*wnt4a*, *wnt10b*, *gsk3*, and *β‑catenin*), is evaluated at defined time points to assess how the material influences inflammatory resolution and pro‑regenerative signaling (Bragato et al., 2024a).

**Figure 1:**
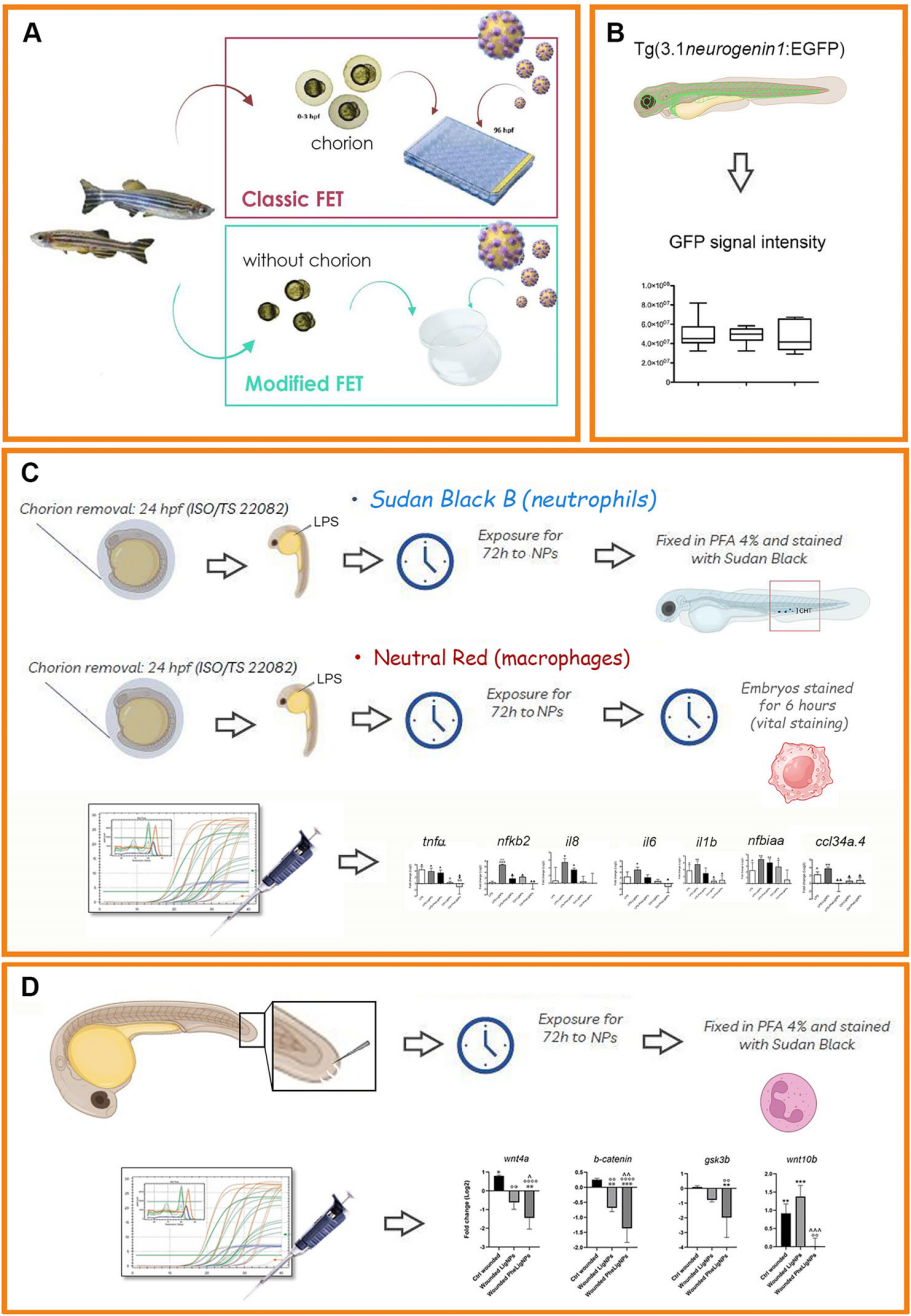
Modular zebrafish embryo platform to profile nano(bio)materials. **(A)** Classic fish embryo acute Toxicity (FET) test on chorionated embryos and modified FET on chemically dechorionated embryos are used to assess developmental compatibility of engineered nanomaterials. **(B)** Neurodevelopmental effects are evaluated in Tg(‐3.1*neurog1*:EGFP) embryos by quantifying GFP signal intensity as a live readout of primary neurogenesis. **(C)** Inflammation is induced by yolk injection of lipopolysaccharide (LPS) in dechorionated embryos, followed by exposure to nanoparticles, Sudan Black B staining to visualize neutrophils or neutral red to observe macrophages, coupled with qRT‑PCR analysis of inflammatory genes (e.g., *tnfα*, *nfkb2*, *il8*, *il6*, *il1β*, *nfkbiaa*, and *ccl34a.4*). **(D)** Injury‑induced inflammation and regeneration are modeled by caudal fin wounding, with subsequent nanoparticle exposure, neutrophil staining, and qRT‑PCR of cytokines and *Wnt/β‑catenin* pathway markers (*wnt4a*, *wnt10b*, *gsk3b*, *and β‑catenin*) to monitor pro‑regenerative responses.

Together, these modules form an integrated pipeline that can be applied sequentially or in different combinations, according to the experimental objectives (**Figure** **1**). A typical implementation might begin with standard FET and morphometrics to establish a sublethal concentration range for a given NM, followed by dechorionated exposures and imaging zebrafish transgenic embryos for more sensitive neurodevelopmental assessment. Once a working concentration is defined, the same conditions can be used to probe immunomodulatory and pro‑ or anti‑regenerative properties, also considering LPS injection, caudal fin wounding, shared qPCR panels, and neutrophil or macrophage staining. By harmonizing exposure conditions, developmental stages, and readouts across modules, this pipeline allows comparison of silica and other nanomaterials with intrinsic physicochemical properties and thus potentially different embryotoxic, neurotoxic, and inflammatory capacity. In conclusion, this zebrafish‐based testing strategy—designed to be accessible to most laboratories— can support the Safe and Sustainable by Design (SSbD) framework and its associated methodologies (Garmendia et al., 2025), offering a practical approach for the development of innovative nanotechnologies and beyond.

Throughout this manuscript, we use the term “module” to denote each of the four overarching experimental objectives illustrated in Figure 1, and the term “protocol” to refer to each specific laboratory procedure. The number of protocols (five) exceeds the number of modules (four) because the inflammation module (Module 3) is addressed by three complementary procedures—Sudan Black B staining, neutral red staining, and LPS microinjection (Basic Protocols 2–4)—each of which can be used independently or in combination depending on the experimental question. The neurodevelopmental module (Module 2), by contrast, is assessed by live imaging of Tg(‐3.1*neurog1*:EGFP) embryos rather than by a dedicated wet lab procedure; this is why the five laboratory protocols map onto four conceptual modules.

The five basic protocols presented in this article include the following: Basic Protocol 1 describes the preparation and suspension of nano(bio)materials together with a pronase‐based embryo dechorionation procedure to enable direct nanoparticle–tissue contact under fish embryo acute toxicity (FET) conditions; Basic Protocol 2 outlines the Sudan Black B staining workflow for fixation, staining, and image‐based quantification of neutrophil recruitment in wild‐type zebrafish embryos; Basic Protocol 3 provides the neutral red vital staining procedure and imaging steps for in vivo visualization of macrophage distribution and activity; Basic Protocol 4 describes the preparation of *Pseudomonas aeruginosa* LPS solutions and the yolk sac microinjection procedure to generate a standardized acute inflammatory model; and Basic Protocol 5 describes the caudal fin wounding procedure used to model injury‐induced inflammation and tissue regeneration.

*NOTE*: All protocols involving animals must be reviewed and approved by the appropriate Animal Care and Use Committee and must follow regulations for the care and use of laboratory animals. Appropriate informed consent is necessary for obtaining and using human study material.

## Basic Protocol 1 NANOPARTICLE PREPARATION AND SUSPENSION, AND EMBRYO DECHORIONATION PROCEDURE

Prepare the nanoparticle stock solution and resuspend in Milli‐Q water to a final concentration of 1 mg/ml. For all experimental procedures, the working concentration is obtained by diluting the stock solution in embryo solution (ES) or in 1× N‐phenylthiourea (PTU) diluted in ES solution. PTU is used to inhibit melanophore and iridophore activity, maintaining embryo transparency during staining experiments.

### Materials

Nanoparticle stock solution
Embryo solution (ES; see recipe)
N‐phenylthiourea (PTU), 10× (see recipe)
PTU, 1× (see recipe)
Pronase from Streptomyces griseus (EC 3.4.24.4, CAS 9036‐06‐0, Sigma‐Aldrich)
Fertilized embryos

Petri dishes
Leica M205FA stereomicroscope with DFC450C digital camera and Leica software (Leica)
Malvern Zetasizer S90 (Malvern Instruments Inc., UK) for DLS/zeta potential
Leica M205FA stereomicroscope with DFC450C digital camera and Leica software (Leica)
GraphPad Prism 9.2.0.332 (GraphPad Software) for statistics

#### Pronase solution protocol

1 The enzyme needs to be resuspended before use. Resuspend 1 g of pronase powder in 4 ml of bi‐distilled water. Divide the resuspended pronase into 100 µl aliquots (250 mg/ml). Store aliquots at −80°C.
2 The stock solution (A) at a concentration of 10 mg/ml can be obtained by adding 2.4 ml of ES to 100 µl of pronase solution. Store at −20°C until use.
3 To obtain a working solution (B) at a concentration of 1 mg/ml, further dilute 2.5 ml of solution A with ES at 1:10 to a final volume of 25 ml (2.5 ml + 22.5 ml ES).

#### Dechorionation procedure

4 Prepare and warm up solution B to approximately 26°C.
5 Collect and select fertilized embryos.
6 Incubate the 6 hpf developmental‐stage‐selected embryos with pronase in a glass Petri dish (or, for embryos collected before completion of epiboly, an agarose‐coated dish to minimize yolk rupture; see Critical Parameters) for 30 s at room temperature (RT), through gentle shaking under a stereomicroscope.
7 Wash the embryos at least 6 or 7 times with ES. If some chorions are still intact, mechanical rupture must be performed using needles.
8 Recover the embryos and expose them to nanoparticles at decided concentrations.
  Note: When using pronase, all materials used (Petri dishes and Pasteur pipettes) must be made of glass. For embryos dechorionated before completion of epiboly, an agarose‐coated glass dish provides the recommended soft surface (see Dechorionation procedure and Critical Parameters). Only after pronase elimination can the plastic materials be used.

## Basic Protocol 2 SUDAN BLACK B STAINING PROTOCOL

Thanks to the zebrafish embryo transparency, it is possible to observe movements and migrations of immune system cells during an innate immune response, particularly neutrophils, which are the first to be recruited to the damage site (Xie et al., 2021). As reported in the literature, to highlight mature neutrophils, Sudan Black B histochemical staining can be performed (Bennett et al., 2001; Lae Guyader et al., 2008; Yang et al., 2014).

### Materials

Embryos at 96 hpf
Paraformaldehyde (PFA), 4% methanol‐free in PBS (for fixation)
Phosphate buffered saline (PBS)
Sudan Black buffer solution (see recipe)
Sudan Black stock solution (see recipe)
Working solution (see recipe)
PBST solution (PBS + 0.1% Tween20)

Syringe filters, 0.45 µm
Magnetic stirrer

#### Staining Procedure

1 Fix embryos at 96 hpf in 4% buffered paraformaldehyde (PFA) for 2 h at RT (or at 4°C overnight).
2 Wash for 15 min in phosphate buffered saline (PBS) (2 times).
3 Wash 5 min (3 times).
4 Incubate embryos in Sudan Black B for 20 min at RT.
5 Wash with ethanol (EtOH) at different concentrations:
  10 min with 70% EtOH + 30% water (3 times)
  10 min with 50% EtOH + 50% PBST (2 times)
  10 min with 25% EtOH + 75% PBST (2 times)
6 Wash with PBST solution (PBS + 0.1% Tween20) for 5 min (2 times).

## Basic Protocol 3 NEUTRAL RED STAINING

Macrophages can be visualized in vivo in zebrafish embryos using neutral red, a vital dye that accumulates in their acidic phagolysosomes; building on foundational characterization of zebrafish macrophage biology, this approach has been widely applied to study macrophage populations, including their responses to NMs (Duan et al., 2017; Herbomel et al., 2001).

### Materials

Neutral red solution (see recipe)
Milli‐Q H_2_O
Embryos
Bio‐based NMs of interest diluted in 1× PTU
Embryo medium (EM)
TRIzol reagent (15596018, Invitrogen) for RNA extraction
Ethyl 3‐aminobenzoate methanesulfonate salt (Tricaine)

Petri dishes
Eppendorf tubes, 2 ml
Glass wells, 60 mm
Stirring plate
Leica M205FA stereomicroscope with DFC450C digital camera and Leica software (Leica)
LunaScript RT SuperMix Kit (New England Biolabs) for cDNA synthesis
Phusion High‐Fidelity DNA polymerase (Finnzymes, Thermo Fisher Scientific) for PCR amplification
Luna Universal qPCR Master Mix (New England Biolabs)
Quantum 3 Real‐Time PCR system (Applied Biosystems)
GraphPad Prism 9.2.0.332 (GraphPad Software) for statistics

#### Staining procedure

1 Select the embryos and let them grow at 28°C in a Petri dish.
2 When the embryos reach 30 hpf, expose them to different concentrations of the bio‐based NMs of interest diluted in 1× PTU for at least 60 h.
3 Check the embryos and separate them (n = 10 for staining, at least n = 10 for RNA extraction).
4 Collect the embryos for the RNA extraction into a 2‐ml Eppendorf tube, into the medium in which they are growing.
5 Collect the embryos for staining into a 60‐mm‐diameter glass well.
6 Expose the embryos diluted in EM at 28°C for 5.5–6 h to neutral red staining solution (2.5 ug/ml; see step 1), shaded with an aluminum foil.
7 After 5.5–6 h, place the embryos in TRIzol for RNA extraction. These can be stored at −80°C to be processed later.
8 Wash 5 times with EM on a stirring plate.
9 Anesthetize embryos in tricaine.
10 Start photographing the embryos under a stereomicroscope.
11 Repeat for all the experimental conditions to be observed

#### Image acquisition

Following neutral red staining, each embryo needs to be photographed using a stereomicroscope to observe macrophages. Based on the number of concentrations to be tested, the embryos can be taken outside the incubator (at a temperature lower than 26°C) to slow down the development.

## Basic Protocol 4 LPS MICROINJECTION PROTOCOL

To generate the first inflammation model, bacterial LPS from *Pseudomonas aeruginosa* was used. Like bacterial endotoxins, LPS represents a potent means for inducing an inflammatory state capable of stimulating an immune response (Novoa et al., 2009).

### Materials

LPS solution (see recipe)
30 hpf embryos
0.016% ethyl 3‐aminobenzoate methanesulfonate salt (Tricaine) for anesthesia
Nanoparticles

Agarose gel mold
Malvern Zetasizer S90 (Malvern Instruments Inc., UK) for DLS/zeta potential
Leica M205FA stereomicroscope with DFC450C digital camera and Leica software (Leica)
GraphPad Prism 9.2.0.332 (GraphPad Software) for statistics

#### Microinjection procedure

1 When the embryos reach the 30 hpf developmental stage, they can be used.
2 Anesthetize embryos with a medium containing 0.016% ethyl 3‐aminobenzoate methanesulfonate salt and place them on an injection mold made of agarose gel.
3 Inject anesthetized embryos into the apical portion of the yolk sac with 1 nl of LPS solution (B) using a microinjector.
4 Recover the injected embryos at 28.5°C and expose them to nanoparticles at the desired concentration.
5 Monitor the injected embryos regularly every 24 h.

## Basic Protocol 5 WOUND INFLAMMATION MODEL GENERATION PROTOCOL

For the wound inflammation model, a wound needs to be created at the terminal portion of the caudal fin of zebrafish embryos at 48 hpf. This is a widely used technique to generate inflammation models useful for anti‐inflammatory drug development (Page et al., 2013).

### Materials

Freshly sterilized zebrafish embryos
PTU, 1×
0.016% ethyl 3‐aminobenzoate methanesulfonate salt

Agarose bed, 3%
Microinjection needle tips

#### Procedure

1 Select freshly fertilized embryos and incubate them at 28.5°C until the 24 hpf stage.
2 Manually dechorionate the 24 hpf embryos and place them in 1× PTU until the following day.
3 At 48 hpf, anesthetize embryos with 0.016% ethyl 3‐aminobenzoate methanesulfonate salt + 1× PTU, and place on a 3% agarose bed.
4 Using a microinjection needle tip, create small lesions on the embryo's caudal fin.
  Important: The same operator should perform wounding on all embryos in the same manner to ensure experimental reproducibility.
5 Recover and expose the embryos to the desired nanoparticles.

## REAGENTS AND SOLUTIONS

### Embryo solution (ES)

0.1 g sodium bicarbonate (NaHCO_3_)
0.1 g Instant Ocean
0.19 g calcium sulfate (CaSO_4_·2 H_2_O)

### LPS solution

Lipopolysaccharide (LPS) from Pseudomonas aeruginosa (strain ATCC 27316, Sigma‐Aldrich)
Milli‐Q water

Dilute *Pseudomonas aeruginosa* LPS powder in Milli‐Q water to obtain a stock concentration of 1 mg/ml (solution A). Further dilute part of the stock solution to obtain working aliquots at a concentration of 250 µg/ml (solution B), which does not have toxic effects on embryos (Yang et al., 2014)

### N‐phenylthiourea (PTU), 10×

Dissolve 0.3 g of 1‐phenyl‐2‐thiourea (Sigma‐Aldrich) powder in 1liter of bi‐distilled water.

### Neutral red solution

Weigh 10 mg of neutral red powder (Sigma‐Aldrich) and dilute it in 10 ml of Milli‐Q H_2_O. The solution must be sheltered from light with aluminum foil and stored at room temperature for 3 months.

### PTU, 1×

Dilute 10× PTU in ES.

### Sudan Black buffer solution

Crystalline phenol
Absolute ethanol (EtOH)
Double‐distilled water
Na_2_HPO_4_·12H_2_O

Dissolve 16 g of crystalline phenol in 30 ml of absolute ethanol. Add this solution to 100 ml of double‐distilled water in which 0.3 g of dibasic sodium phosphate (Na_2_HPO_4_·12H_2_O) is already dissolved.

### Sudan Black stock solution

Sudan Black B powder (Sigma‐Aldrich)
Absolute ethanol (EtOH)

Completely dissolve 0.3 g of Sudan Black powder in 100 ml of absolute ethanol. Leave the solution at RT in a magnetic stirrer for 2 days.

### Working solution

Mix 40 ml of buffer solution with 60 ml of Sudan Black solution. Filter using a syringe filter with 0.45‐µm porosity.

## COMMENTARY

### Critical Parameters

Critical parameters across these protocols relate primarily to maintaining consistent embryo handling, reagent stability, and staining conditions so that subtle nano(bio)material effects can be distinguished from technical artefacts. For developmental exposures and dechorionation, it is essential to use freshly prepared PTU (≤3 months) to avoid increased baseline cardiac edema in controls, and to standardize pronase concentration and exposure time to achieve efficient chorion removal without excessive mortality. For laboratories working with embryos younger than 10 hpf—that is, prior to completion of epiboly—or where maximum baseline viability is a priority, we recommend performing the pronase dechorionation step in agarose‐coated dishes rather than glass, as this prevents yolk rupture caused by contact with hard surfaces and can reduce background mortality. In imaging‐based assays, careful control of staining duration and destaining steps is crucial. Sudan Black B–stained embryos should be checked under a stereomicroscope before ethanol washes, and if the signal is weak, they should be kept a few minutes longer in the stain to prevent poor neutrophil labeling. In contrast, neutral red–stained embryos may require closer monitoring for around 5.5–6 h to ensure adequate macrophage signal without excessive background. For working with fluorescent transgenic lines, genetic background and fluorescence intensity must be managed by crossing transgenic fish with wild‐type partners when needed, thereby normalizing baseline fluorescence and enabling detection of modest exposure‐related differences.

### Troubleshooting Table

The troubleshooting table summarizes common issues encountered during staining, dechorionation, and imaging (e.g., weak Sudan Black or neutral red signal, increased baseline edema, or overly bright transgenic fluorescence), links them to likely procedural causes, and provides practical adjustments to restore robust, interpretable readouts (Table 1).

**Table 1:** Troubleshooting Guide Useful for the Procedures

Problem	Possible cause	Solution
Sudan Black— poor signal	Washed with EtOH too soon	Check the embryos under a stereomicroscope at the end of the 20th staining. If they are not colored enough (they might be dark blue), proceed with the staining for a few more minutes.
Neutral red— poor signal	Embryo staining needed for a longer time	Check the embryos frequently around 5.5 h after starting the staining.
Increased number of control embryos presenting cardiac edema (more than 1 %)	10× PTU stock solution older than 3 months	Prepare a new 10× PTU stock solution.
Difficulty in observing fluorescent signal differences in transgenic embryos (green or red signal too bright to be evaluated in exposed embryos compared to controls)	Problems related to the initial screening of transgenic embryos; reduced instrumentation resolution	To reduce the fluorescence and be able to evaluate even slight differences between exposed embryos and controls, breed transgenic zebrafish (female) with wild‐type zebrafish (male). This will normalize fluorescence for the experiment.

### Understanding Results

When these protocols are executed correctly, the combined readouts from FET, dechorionated exposures, inflammatory challenges, and regeneration assays should converge into a coherent profile of nano(bio)material compatibility and immunomodulatory activity in zebrafish embryos, as schematized in Figure 1.

In Basic Protocol 1 (nanoparticle preparation, suspension, and embryo dechorionation), users should first verify that nanoparticle stock and working suspensions are macroscopically homogeneous and remain stable over the exposure period. In chorion‐intact FET assays, a compatible nano(bio)material typically causes low mortality (≤10%–15% at the highest test concentrations) and only scattered, non‐dose‐dependent malformations; quantitative morphometrics at 96 hpf (body length, eye area/diameter, interocular distance, and yolk sac area) should remain within roughly ±10% of vehicle controls. After pronase‐based dechorionation, a background mortality of ∼20%–25% is expected and should be comparable between control and exposed groups; under these conditions, truly adverse materials may reveal subtle growth delay or edema phenotypes that were not evident with intact chorions, whereas bio‑based silica and lignin nanoparticles that are largely biocompatible continue to show minimal effects on survival and gross morphology.

In Basic Protocol 2 (Sudan Black B staining), high‐quality staining yields sharply defined, dark myeloperoxidase‐positive neutrophils on a low‐background tissue field after ethanol destaining. In non‑wounded controls, neutrophils should be concentrated in hematopoietic regions such as the caudal hematopoietic tissue, with low variability within a group. After caudal fin wounding or LPS challenge, users should observe a reproducible increase in Sudan Black–positive cells at the wound site or perivascular regions, which can be quantified as enlarged positive area or increased particle counts within a standardized ROI in Fiji/ImageJ; in our experience, bio‑based LigNPs and PheLigNPs at subtoxic doses do not further enhance neutrophil recruitment beyond that induced by the primary inflammatory stimulus, indicating that they do not exacerbate acute inflammation at the tissue level.

In Basic Protocol 3 (neutral red staining), correctly stained embryos show discrete neutral red–positive macrophages in expected anatomical locations (e.g., yolk sac and trunk vasculature), with minimal diffuse background. In control conditions, macrophage number and distribution should be consistent across embryos, whereas strongly immunostimulatory or cytotoxic nano(bio)materials may increase macrophage accumulation or alter their overall pattern. In our experiments (paper in submission), neutral red staining patterns were comparable to the inflammatory picture obtained with *Pseudomonas aeruginosa* LPS (positive control for inflammation), and this cellular observation is aligned with qPCR data distinguishing pro‑inflammatory gene expression signatures (e.g., *ccl34a.4* gene expression level significantly increased).

In Basic Protocol 4 (LPS microinjection), a well‐executed series is characterized by minimal mechanical damage from yolk injections and high survival in vehicle‐injected controls, alongside a robust, dose‑appropriate inflammatory response in LPS‑injected embryos. At 24–48 h after microinjection of *Pseudomonas aeruginosa* LPS (1 nl of 250 µg/ml), users should detect significant upregulation of inflammatory genes such as *tnfα*, *nfkb2*, *il8*, *il6*, *il1β*, *nfkbiaa*, and *ccl34a.4* in qRT‑PCR assays, and when combined with the staining protocols above, an increased recruitment of neutrophils and context‑dependent changes in macrophage behavior. Nano(bio)materials with mild or beneficial immunomodulatory profiles are expected to modulate this response only modestly (e.g., slight attenuation or reshaping of cytokine expression without worsening pathology), whereas strongly pro‑inflammatory materials may further amplify cytokine levels and inflammatory cell recruitment compared to LPS‑only controls.

For Basic Protocol 5 (wound Inflammation model generation), correctly wounded embryos at 48 hpf show a reproducible cut in the caudal fin with preserved overall morphology and high survival in all groups. In negative controls (wounded but not exposed to test materials), users should observe a robust yet spatially confined inflammatory response, characterized by neutrophil and macrophage recruitment to the wound margin over 24–72 h, accompanied by progressive tissue regrowth when quantified by fin area. Nano(bio)materials that do not exacerbate acute damage will leave wound‑induced inflammation and regeneration kinetics largely unchanged, whereas highly pro‑inflammatory or toxic materials may delay regrowth, increase edema, or distort the temporal profile of inflammatory cell infiltration.

Across all modules, high embryo‑to‑embryo variability within a condition, unstable morphometric baselines, inconsistent neutrophil or macrophage counts, or noisy gene expression profiles generally signal technical issues such as poorly dispersed nanoparticles, inconsistent dechorionation, variable injection volumes, or suboptimal fixation and staining. Including both negative controls (vehicle, non‑wounded, and non‑injected) and positive controls (standardized LPS injection and caudal fin wounding) in each experiment is therefore essential, allowing users to benchmark their data against the module‑level expectations summarized in Figure 1 and the reference outcomes previously obtained for silica‐ and lignin‐based nanoparticles in zebrafish embryos.

### Time Considerations

In Basic Protocol 1 (nanoparticle preparation, suspension, and embryo dechorionation), starting from the nanoparticle preparation to obtain pronase‐chorion‐deprived embryos that can be exposed to the NPs or NMs, it will take approximately 2.5 h.

In Basic Protocol 2 (Sudan Black B staining), starting from the 4% PFA fixation to the time of imaging, the time estimated is 4 h.

In Basic Protocol 3 (neutral red staining), the duration of this vital staining is 5.5–6 h. The estimated time of imaging depends on the number of embryos that need to be imaged.

In Basic Protocol 4 (LPS microinjection), the time needed depends on the number of embryos that need to be injected. Preparing the LPS aliquots before starting the protocol (5 µl, store at – 20°C) helps accelerate the operation.

In Basic Protocol 5 (wound inflammation model generation), the embryos at 48 hpf need to be anesthetized in tricaine and wounded with a needle under a stereomicroscope. Time estimated: 1.5 h.

### Author Contributions

**Cinzia Bragato**: Conceptualization; investigation; writing—original draft; methodology; validation; visualization; writing—review and editing; software; formal analysis; data curation; supervision. **Alessandra Lama**: Investigation; formal analysis. **Greta Carbone Faccin**: Investigation. **Johan George Varghese**: Investigation. **Rossella Bengalli**: Investigation; writing—review and editing. **Patrizia Bonfanti**: Investigation; writing—review and editing. **Anita Colombo**: Writing—original draft; writing—review and editing. **Maurizio Gualtieri**: Writing—review and editing; data curation. **Paride Mantecca**: Problems related to the initial screening of transgenic embryos; reduced instrumentation resolution; funding acquisition; writing—review and editing; resources; project administration.

### Conflict of Interest

All authors declared that there are no conflicts of interest.

## Supporting information

Supporting Information

## Data Availability

The data, tools, and materials (or their source) that support the protocol are available from the corresponding author upon reasonable request.
