# Supplementary material for: Zebrafish Embryos to Profile Nano(bio)materials: A Modular Platform for Developmental Toxicity, Neurotoxicity, and Inflammation‐Regeneration Assays
Source: Curr Protoc. 2026 Jun 13;6(6):e70403. doi: 10.1002/cpz1.70403 (PMC13263913; doi:10.1002/cpz1.70403)
Supplement: Supplementary file 1 — Supporting Information [file CPZ1-6-0-s001.docx]

**Supplementary Information** - Primer sequences of genes evaluated for inflammation and Oxidative Stress.

| **INFLAMMATION**  **And OXIDATIVE STRESS** | **FORWARD (5’🡪 3’)** | **REVERSE (5’🡪 3’)** |
| --- | --- | --- |
| *tnfα* | CGCTGGTGATGGTGTCTAGG | CCCTGGGTCTTATGGAGCGT |
| *Il8* | GTCGCTGCATTGAAACAGAA | CTTAACCCATGGAGCAGAGG |
| *il6* | GGCATTTGAAGGGGTCAGGA | GCGTTAGACATCTTTCCGTGC |
| *il1β* | CGTACTCAAGGAGATCAGCGG | GCGGTGCTGATAAACCAACC |
| *nfkbiaa* | ACAACCGAAGAGAGAACATGGA | CGAAATCTCCCGCGTCTCAT |
| *ccl34a.4* | CTTTGACGCATGGAGGATTT | TGCAGCTCAACCAGAAGATG |
| *nfkb2* | TGGCTGGAGCACTAAGGATG | CCTCTCTGCTTTGGCTCCTC |
| *cxcl-cic* | CATCCGGCCAGCTCTGCTTGAAT | CCACTCTTGACCTCCTGTGCTCTCT |
| *hmox1a* | CCACGTCAGAGCTGAAAACA | AGCGCTCGGTAGATCTCGTA |
| *nrf2a* | GAGCGGGAGAAATCACACAGAATG | CAGGAGCTGCATGCACTCATCG |
| *nrf2b* | GGCAGAGGGAGGAGGAGACCAT | AAACAGCAGGGCAGACAACAAGG |
| **REGENERATION** |  |  |
| *wnt4a* | CAATGCGAGCAACTGGCTATAC | AATGCAGCTTCCCTCGTACCTT |
| *gsk3β* | TCTGCTCACCGTTTCCTTTC | CTCCGACCCACTTAACTCCA |
| *β-catenin* | GGAGCTCACCAGCTCTCTGT | TAGCTTGGGTCGTCCTGTC |
| *wnt10b* | TCCTGAAACAGGCTCGAAGT | GCTGCTCACTTGCACACA |
| **HOUSEKEEPING** |  |  |
| *Ism12b* | AGTTGTCCCAAGCCTATGCAATCAG | CCACTCAGGAGGATAAAGACGAGTC |
| *mobk13* | CACCCGTTTCGTGATGAAGTACAA | GTTAAGCAGGATTTACAATGGAG |
